# Reproductive Losses and Their Causes in Alpacas—A Survey-Based Study

**DOI:** 10.3390/ani12213030

**Published:** 2022-11-03

**Authors:** Joanna Kapustka, Monika Budzyńska

**Affiliations:** Department of Animal Ethology and Wildlife Management, University of Life Sciences in Lublin, Akademicka 13, 20-950 Lublin, Poland

**Keywords:** alpaca, reproduction, cria survivability, questionnaire

## Abstract

**Simple Summary:**

Alpaca breeding is becoming increasingly popular in regions other than South America (the place of origin of the species), e.g., in North America and Europe. It is highly important to monitor reproductive disorders and to identify the causative factors of the problem as the profitability of alpaca breeding depends on satisfactory reproductive results. The analysis of the frequency and causes of reproductive problems in alpacas was carried out with the use of questionnaires completed voluntarily by alpaca owners in three regions (British Isles, Continental Europe, and North America). Cases of miscarriage and stillbirths were reported from about 1/3 of the farms, and in half of them, the cases of crias (young up to 6 months of life) and young alpacas fell within 12 months after birth occurred. Preterm births were reported from almost half of the farms. An important element increasing the cria survival rate of is the high birth weight and dams’ mineral supplementation. This study has indicated a relationship between fetal death and the occurrence of infectious diseases and scabies infestation in the herd. They may be potential causes of reproduction-related problems that are not discerned in time.

**Abstract:**

The profitability of alpaca breeding depends on satisfactory reproductive results. The study aimed to analyze the frequency and causes of reproduction-related problems in alpacas, in particular miscarriages, stillbirths, preterm births, twin pregnancies, and falls of crias within 12 months after birth. This analysis was carried out with the use of questionnaires completed voluntarily by 109 alpaca owners in three regions (British Isles, Continental Europe, and North America). Cases of miscarriage and stillbirths were reported from 44% and 36% of the farms, respectively. In half of the farms, the cases of falls of crias and young alpacas within 12 months after birth were reported. Preterm births were reported from almost half of the farms. A large number of alpaca owners did not identify the cause of fetal death. An important element increasing the cria survival rate of is the birth weight (the higher cria birth weight, the lower risk of perinatal complications and less necessity of bottle feeding). Crias from dams receiving mineral supplements had higher birth weight. This study indicated a relationship between fetal death and the occurrence of infectious diseases and scabies infestation in the herd. They may be potential causes of reproduction-related problems that are not discerned in time.

## 1. Introduction

Alpaca (*Vicugna pacos*) breeding is becoming increasingly popular in regions other than South America (the place of origin of the species), e.g., in North America and Europe [1]. The profitability of alpaca breeding depends on satisfactory reproductive results; hence, it is highly important to monitor reproductive disorders and to identify the causative factors of the problem. As reported by Wright et al. [2], 1/3 of mated females do not produce offspring. Van Saun [3] states that it could be even higher (more than 40%). The percentage of pregnancies ending in miscarriage ranges from 7–12% [4] to 15–25% [3]. Moreover, from 9 to 12% of neonates die before reaching 6 months of age [5].

Alpacas, i.e., representatives of the camelid family, have slightly different reproductive physiology than other species of farmed animals. They do not have a typical estrus cycle. The acceptance of the male by the female and mating are dependent on the presence of mature follicles in the ovary [6]. Mating in camelids takes place in a squatting position [6]. Ovulation is induced by copulation and occurs approximately 24–36 h after mating [7,8]. LH preovulatory surge is due to the action of the beta nerve growth factor [9]. Three stimuli are required for a pre-ovulatory surge of LH from the brain: the physical stimulation of the cervix by the male’s penis, the action of chemical compounds contained in the semen, and the characteristic sounds produced by the male during courtship and copulation [7,8]. A pregnant female or a female with no mature follicles in the ovary will not allow the male to mate, but will spit and kick the male [10].

Females at ca. 15–18 months of age and with the minimum body weight of 40 kg can be allowed to reproduce [8]. However, sometimes one-year-old females with the appropriate body weight are mated, as reported by Vaughan and Tibary [11]. In turn, male alpacas reach reproductive maturity at the age of ca. 2.5 years [5]. A correlation has been found between the size of testes and the effectiveness of fertilization [8,12,13]. Pregnancy in alpacas is detected via determination of the blood progesterone level from day 12 (an increase after fertilization), and the first ultrasound examination on ca. day 28 after mating [8]. The changes in behavior of the female towards the male (the female spits and kicks and does not allow mating) [8,12] can be also observed, however they are not a tool to perform pregnancy diagnosis. Gestation in alpacas lasts on average for 335–350 days [6,14]. The parturition progresses with no complications in most cases [15]. It should last for maximum 60 min and the placenta should be expelled within 4–6 h. A placenta that is not expelled at 24 h postpartum is regarded as retained and veterinarian assistance is required [8]. The neonate, called a cria, is born fully functional and is covered by a hair coat. Most labors require little or no help from the breeder. During normal labor, the fetus is in the anterior presentation of the head and forelimbs, and neither of the forelimbs is flexed [8,12]. The causes of perinatal cria deaths may include improper presentation of the fetus during parturition [8], congenital defects [16], or bacterial and parasitic infections [17]. Female alpacas do not lick but usually sniff and touch their neonates with their lips. The cria dries in the sun or is dried off by the breeder. After a few or several tens of minutes, the cria starts to stand up and seeks the udder. It is extremely important for all mammals to ingest immunoglobulin-containing colostrum immediately after birth, as it provides the neonate with immunity in the first period of life. Ingestion of insufficient colostrum amounts or failure to receive it substantially impairs the function of the cria immune system and makes the animal highly susceptible to various infections [18]. During the subsequent days of life, the neonates should gradually increase their body weight [19].

However, pregnancy and parturition do not always proceed without complications. Abnormalities posing potential danger to the cria and the dam may occur both during pregnancy and in the perinatal period. Miscarriages can be caused by genetic (congenital defects) [8] and environmental, non-infectious (maternal, fetal, nutritional, iatrogenic) and infectious (bacterial, viral, fungal, parasitic), factors [4]. As reported by Bravo [7], the first month of pregnancy is the most important period, as the embryo may often be resorbed at that time or may develop further. Pearson et al. [4] have indicated that early miscarriage refers to pregnancies in which the embryo dies before the 50th day of pregnancy. Lethal genes that prevent further development of the embryo are often the cause of embryo resorption. The investigations conducted by Jackling et al. [20] and Jones et al. [21] have demonstrated that there are no classic gray alpaca homozygotes among alpacas, which suggests involvement of a lethal mutation. First-trimester miscarriage is associated with expulsion of the dead fetus but is not accompanied by any evident symptoms in the female. Miscarriage in late pregnancy is associated with clinical symptoms in the dam before the expulsion of the fetus (anxiety, colic, enlarged mammary gland) [4]. The non-infectious causes of pregnancy loss include immaturity of the female (body weight below 25 kg) and poor health status, which is usually observed in extensively reared herds [4]; on the other hand, obesity has a negative effect on reproduction as well [22]. One of the nutritional factors is the deficiency or excess of some macro- and microelements and vitamins (phosphorus, copper, zinc, selenium, and vitamins A and E) [22]. The energy demand increases substantially during the last three months of pregnancy, and supplementation with certain minerals or vitamins should be introduced from the beginning of pregnancy or even earlier [3]. McKenzie et al. [23] have reported a case of miscarriage in an alpaca as a result of poisoning with nitrates and nitrites contained in oat hay. Many illnesses, especially parasitic and infectious diseases, can cause miscarriages and stillbirths, which is a substantial loss for the breeder. Special care should be taken during disposal of the dead fetus and placenta, as there is a risk of infection of the rest of the herd if the miscarriage was caused by infectious diseases [4].

Alpacas usually produce one cria, whereas twins are extremely rare [19]. Live-born twins are usually very weak or do not survive the delivery. Typically, a twin pregnancy is diagnosed during the first ultrasound scan, and later only one fetus may be visible [24,25], but it frequently dies [25]. Occasionally, one of post-miscarriage fetuses is fresh while the other is in an advanced stage of autolysis [4]. Preterm birth (before 335 days of gestation) is another indicator related to the reproduction and rearing of cria. Preterm cria have low birth weight, non-erupted incisors, floppy or bent ears, poor suckling reflex, delicate hair coat, deformed wrist joints, and rubbery caps on toe nails [15,16]. In turn, prolonged pregnancy (over 370 days) may result from abnormal placenta structure, nutritional deficiencies, or fetal abnormalities; however, induction of labor is risky, as these factors usually retard fetal growth [4].

The aim of the study was to analyze the frequency and causes of reproduction-related problems in alpacas, in particular miscarriages, stillbirths, preterm births, and twin pregnancies as well as falls of crias within 12 months after birth.

## 2. Materials and Methods

The analysis of the frequency and causes of reproductive problems in alpacas was carried out with the use of questionnaires completed voluntarily by owners of alpaca farms. The questionnaire was created in Google Forms and made available online for alpaca breeders in countries of continental Europe (Poland, Germany, Belgium, the Netherlands), the British Isles (Great Britain, Ireland) and North America (the USA, Canada). To make the questionnaire available and invite respondents, it was sent directly to the farms’ e-mail addresses, or it was shared on social media on forums dedicated to alpaca rearing. The questionnaire was fully anonymous, and it was not possible to associate any of the responses with a specific keeper/farm. It was produced in two versions: Polish (for farms in Poland) and English (for farms in other countries). It was available for completion from October 2021 to January 2022.

The questionnaire was completed by 109 alpaca owners from the three different regions (22 from the British Isles, 31 from continental Europe, and 56 from North America).

The questionnaire consisted of three parts: (1) farm characteristics (2) animal health status and (3) reproductive indicators (Table 1). The questionnaire form is available in Supplementary Materials. Possible relationships between the farm and health status characteristics and the reproduction indices were determined.

The values of quantitative variables were presented using the mean value and standard deviation. In the case of the qualitative traits, the Chi2 test was used to detect relationships between the analyzed variables. The normality of the distribution of the variables in the studied groups was checked with the Shapiro–Wilk normality test. Student’s *t*-test was used to test the differences between two groups and, when the conditions for the use of this test were not fulfilled, the Mann–Whitney U test was employed. Differences between three or more groups were evaluated using the Kruskal–Wallis test. The Pearson r correlation was also used to determine the relationships between some variables. Statistically significant differences or correlations were indicated at the significance level of *p* < 0.05 and correlations close to statistical significance were indicated at *p* < 0.1. The database and statistical analysis were developed based on Statistica 9.1 computer software (StatSoft, Poland).

## 3. Results

According to the survey results, smaller farms with up to 20 females and a maximum of 5 stud males accounted for 50% of the total number. The greatest number of large farms (over 20 females of the herd) were located in North America (31% of all surveyed farms); small farms in this area accounted for 20% (large farms predominated over small farms only in this region). There were 7% of large farms and 22% of small farms in Central Europe, whereas large and small farms in the British Isles constituted 8% and 12%, respectively. Most farms (58%) were established less than 10 years ago. Other farm animals (cattle, goats, sheep, and horses) are reared in addition to alpacas in a large number of the farms (41%). The birth weight of most cria (56%) was higher than 7 kg. Diagnosis of pregnancy with the use of ultrasound scan was declared by 69% of alpaca owners. Cases of miscarriage and stillbirths in the last 3 years were reported from 44% and 36% of the farms, respectively. In half of the farms, the owners reported cases of falls of crias within 12 months after birth. Most pregnant females received mineral (74%) and/or vitamin supplementation (60%). In 76% of farms, crias had to be bottle fed. Furthermore, 38% of the respondents reported cases of scabies infestation and/or infectious diseases (22%) in the herd. Preterm births were reported from almost half of the farms (48%), with a vast majority of these crias reaching the age of 6 months (78%).

In the questionnaire, twin pregnancies in their herd were reported by 21 alpaca owners (19%). Due to the small size of the group, there were no significant relationships between the independent variables and the occurrence of twin pregnancies. As many as 17 (81%) of the 21 cases ended in miscarriage, neither of the twin crias survived birth in one case, and only one cria was born alive and reached the age of 6 months in two cases. There was only one case of both crias (females) born alive and reaching the age of 6 months.

### 3.1. Factors Related to Farm Characteristics

Significant correlations were found between some characteristics of the farm and herd health status and the selected reproductive indicators.

The analysis of the relationship between the location of the farm and the analyzed indicators was performed using the Kruskal–Wallis test (Table 2). The Mann–Whitney U test demonstrated differences in the mean birth weight of full-term and preterm crias between the analyzed regions. The highest birth weight was recorded in the crias from the British Isles; it differed significantly from the birth weight recorded in North America (*p* = 0.0135). The lowest mean birth weight was exhibited by preterm crias from North America; it differed significantly from the birth weight of crias born in the British Isles (*p* = 0.0006) and continental Europe (*p* = 0.014). There were no significant differences between the frequency of miscarriages, stillbirths, or falls within 12 months after birth and the location of the farm.

**Figure 1:**
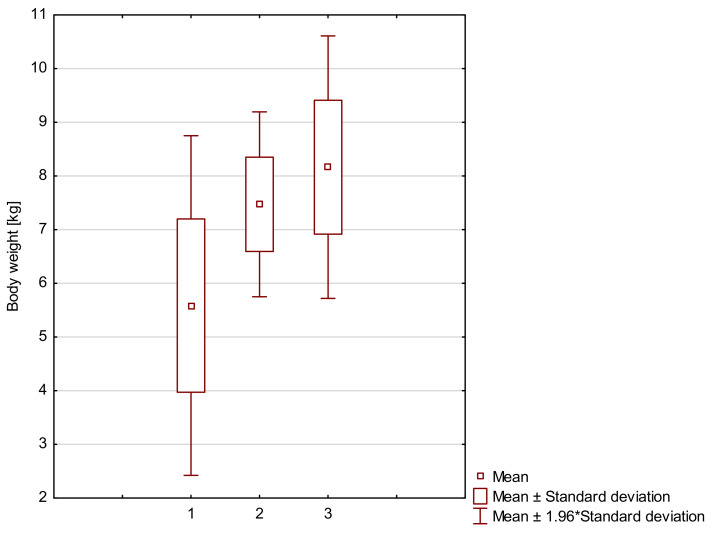
Mean birth weight cria (kg) with the birth term. Numbers denote: 1—mean body weight of premature cria, 2—mean body weight of cria born in term, 3—mean body weight of postmature cria.

Figure 1 shows the mean birth weights of full-term crias recorded in the surveyed farms. As shown by the Mann–Whitney U test, the mean birth weight of preterm crias ($\bar{x}$ = 5.59 kg) was lower (*p* < 0.01) than the mean birth weight of crias born at term ($\bar{x}$ = 7.47 kg). In turn, the mean birth weight of post-term crias was 8.16 kg.

**Figure 2:**
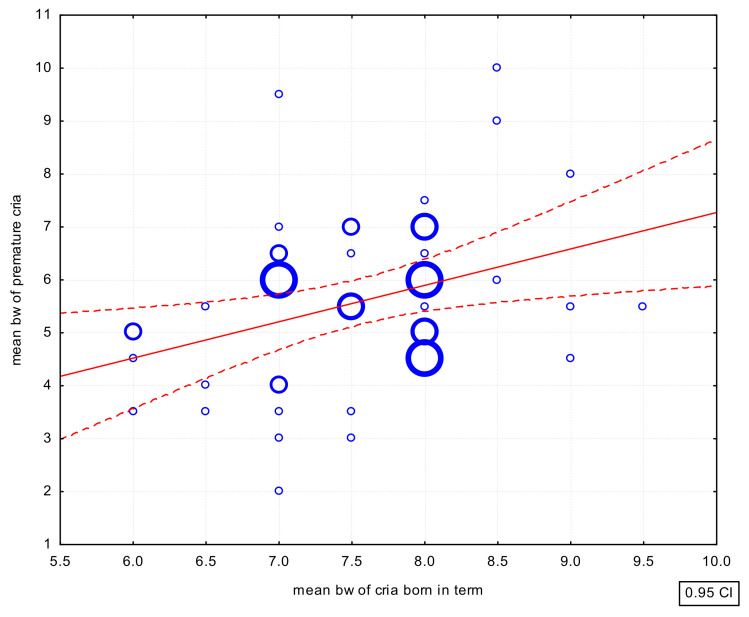
Scatterplot of the correlations of the mean birth weight in full-term and preterm cria. bw—body weight.

**Figure 3:**
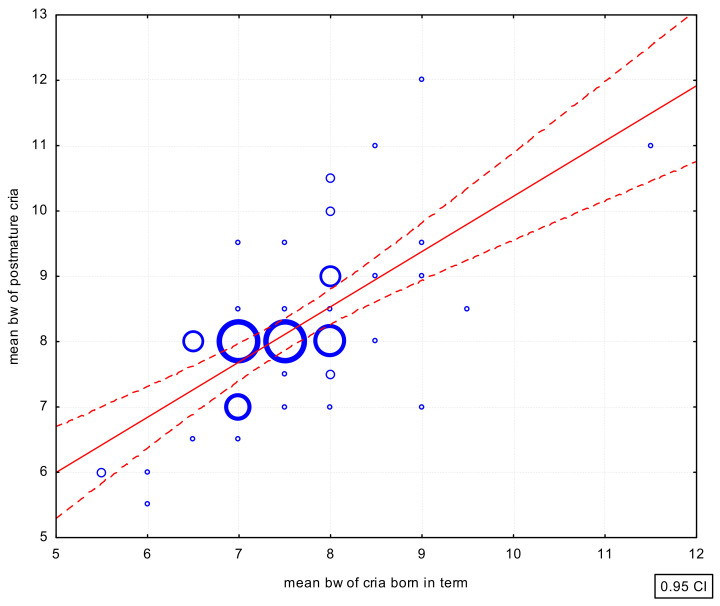
Scatterplot of the correlations of the mean birth weight in full-term and post-term cria. bw—body weight.

There were also positive correlations between the mean birth weight of crias born at term and the mean birth weight of preterm (r = 0.3434; *p* < 0.05) (Figure 2) and post-term (r = 0.6492; *p* < 0.01) crias (Figure 3). However, the correlation in the case of the birth weight of preterm crias was weak.

The Mann–Whitney U test and Student’s t-test (data on the birth weight of preterm crias) revealed significant differences between the duration of running the farm and the birth weight of crias, taking into account the birth term. A significant difference was found between the mean birth weight of preterm crias on farms run for less than 10 years ($\bar{x}$ = 6.13 kg) and over 10 years ($\bar{x}$ = 5.04; *p* = 0.013). In turn, in the group of the post-term crias, these values were $\bar{x}$ = 8.5 kg and 7.67 kg, respectively (*p* = 0.011). Interestingly, there were more cases of stillbirths on the longer-run ($\bar{x}$ = 0.67) than shorter-run farms ($\bar{x}$ = 0.3; *p* = 0.007) in a three-year scale. The mean birth weights of crias born at term were similar on farms run for less than 10 years and those run for a longer time ($\bar{x}$ = 7.55 kg vs. $\bar{x}$ = 7.37 kg, respectively).

The Mann–Whitney U test and Student’s *t*-test (data on the birth weight of preterm crias) demonstrated higher mean birth weight of preterm crias in herds with less than 20 females, i.e., 6.13 kg, than the value of this parameter recorded in the larger herds, i.e., 5.16 kg (*p* = 0.029). There were more cases of miscarriages, stillbirths, and deaths within 12 months after birth in the larger ($\bar{x}$ = 1.56, 0.74, and 2.16, respectively) than smaller ($\bar{x}$ = 0.59, 0.22, 0.76; *p* = 0.002, 0.0001, 0.014) herds.

### 3.2. Factors Related to Herd Health

The survey results highlighted both positive and negative aspects of herd health-related factors that may affect reproduction performance in alpacas.

#### 3.2.1. Dams’ Supplementation

The Mann–Whitney U test showed a relationship between the birth weight of full-term crias and the mineral supplementation in dams during pregnancy (Table 3). Crias from dams receiving mineral supplements had higher birth weight (*p* = 0.0198). Similarly, the mineral supplementation had an effect on the birth weight of post-term born crias (*p* = 0.057). There were no such differences in the occurrence of miscarriages, stillbirths, or deaths within 12 months after birth. Similarly, no significant differences were found between these variables and the vitamin supplementation (Table 3).

#### 3.2.2. Necessity of Bottle Feeding

**Figure 4:**
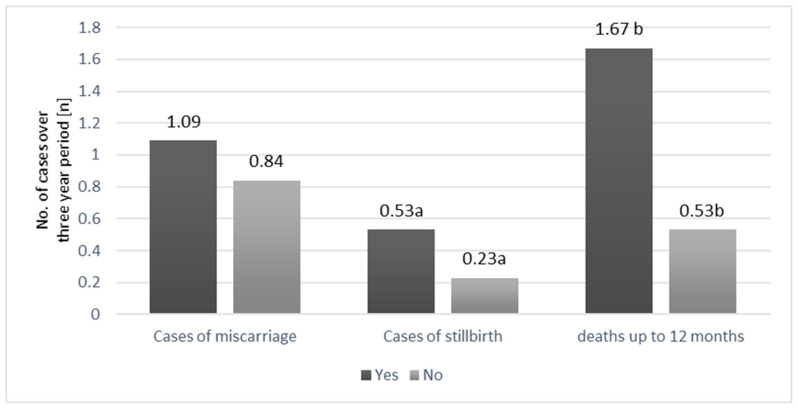
Relationship between the frequency of reproductive losses (n) and the necessity of bottle feeding (yes/no). a, b—values marked with the same letters differ significantly (*p* < 0.05).

The necessity to bottle feed a cria always indicates that the animal is at risk. The present study confirmed the relationship between preterm births and the necessity to bottle feed the crias (Chi2 = 3.926; *p* = 0.048). Moreover, as shown by the Mann–Whitney U test (Figure 4), the percentage of falls within 12 months after birth was higher in the groups of bottle-fed crias (bottle feeding: $\bar{x}$ = 1.67 vs. no bottle feeding: $\bar{x}$ = 0.53, *p* = 0.0157). This group was also characterized by a greater frequency of stillbirths (bottle feeding: $\bar{x}$ = 0.53 vs. no bottle feeding: $\bar{x}$ = 0.23, *p* = 0.045).

#### 3.2.3. Disease Occurrence in the Herd

Scabies infestation was one of the most frequent problems reported by the owners of alpaca herds (almost 4 out of 10 respondents, 37%, indicated current or previous scabies infestations on the farm in the last 3 years). The present study has shown that the health status in the herd, not only the presence of scabies but also other infectious diseases (bacterial, viral, fungal), may have an impact on the reproduction performance of alpacas. There was a relationship between the frequency of preterm births and miscarriages in the herd and scabies infestations (Chi2 = 8.676, *p* = 0.0032 and Chi2 = 3.879, *p* = 0.0489, respectively). Moreover, a relationship was found between the occurrence of scabies or infectious diseases and the indication of an unknown cause of miscarriages by the questionnaire respondents (Chi2 = 3.514, *p* = 0.061 and Chi2 = 7.503, *p* = 0.0062, respectively) and between the occurrence of infectious diseases and an unknown cause of stillbirths (Chi2 = 6.748, *p* = 0.0094). There was no such relationship with regard to the other causes of miscarriages or stillbirths (e.g., severe maternal stress, weather factors, or congenital defects) (0.918 > *p* > 0.123). Additionally, there was no correlation between the presence of other farm animals (e.g., cattle, sheep, goats, horses) and the prevalence of infectious diseases in the herd (Chi2 = 0.437; *p* = 0.509). The differences between the analyzed variables presented in Table 3 were assessed with the use of the Mann–Whitney U test. In correlation with the presence or absence of infectious diseases in the herd, there were significant differences in the birth weight of crias born at term (disease in the herd: $\bar{x}$ = 7.92 kg, no disease in the herd: $\bar{x}$ = 7.35 kg; *p* = 0.017), cases of miscarriages (disease in the herd: $\bar{x}$ = 1.71, no disease in the herd: $\bar{x}$ = 0.85; *p* = 0.0006), cases of stillbirths (disease in the herd: $\bar{x}$ = 0.92, no disease in the herd: $\bar{x}$ = 0.33; *p* = 0.0009), and cases of falls within 12 months after birth (disease in the herd: $\bar{x}$ = 2.88, no disease in the herd: $\bar{x}$ = 0.99; *p* = 0.019) (Table 4).

## 4. Discussion

Rearing conditions undoubtedly exert a significant impact on the indicators of animal reproduction. Alpacas have atypical reproductive physiology with an essential role of the male in triggering ovulation. In these animals, the pregnancy is long, only one cria is born, and the fertility rate is low. Hence, in the case of these extremely valuable animals, any measures should be taken to prevent miscarriages and offer crias the best possible start in life.

The cria born before term (before 335 days of gestation) had significantly lower birth weight than those born at term. As shown by Tibary et al. [15], the weight of a preterm neonate can be over 20% lower than that of a full-term cria. Similar results were reported by Hardefeldt et al. [26] in their study conducted in the USA. The mean birth weight of preterm crias recorded in their study was 6.5 kg, while full-term animals weighed 8.8 kg. Davis et al. [1] demonstrated that the mean cria birth weight depended on the season and ranged between 7.8 and 8.8 kg. As indicated by Tibary et al. [15], the minimum cria birth weight considered to be normal is 7 kg. In the present study, the birth weight of preterm crias reported from North America was 4.53 kg. The birth weight of full-term crias was 7.34 kg, which was lower than the value of this parameter in continental Europe ($\bar{x}$ = 7.42) or the British Isles ($\bar{x}$ = 7.86), where the heaviest crias were born. This may be related to the fact that the alpaca maintenance system in North America is slightly more extensive than in Europe and the herds are larger (a greater number of North American respondents declared having a herd of more than 20 animals, whereas the number of smaller herds was almost equal in the three analyzed regions), which limits the possibility of individualized care of each animal. Preterm delivery may be caused by a variety of factors, e.g., infectious diseases, maternal stress, poor health status, and unfavorable weather conditions. As reported by Whitehead [16], maternal stress at the end of pregnancy (related to, e.g., shearing, transport, vaccinations, deworming) and unfavorable weather conditions (e.g., high temperature and humidity) increase the risk of preterm birth. Bacterial infections can cause preterm births as well [26,27,28,29]. In the present study, no significant correlation was found between the specific cause and the higher prevalence of preterm births; however, crias from herds infested by scabies had lower birth weight. 

Additionally, preterm birth was usually associated with the necessity to bottle feed the crias. Low birth-weight, preterm, or immature crias may be at risk of disorders related to passive transfer of immunoglobulins from the gastrointestinal tract [15,16] and perinatal infections or hypoxia [15], which may even be the leading causes of perinatal deaths. The best solution is to provide colostrum from the same species. However, the dam does not always will to be milked [19]. In this case the alpaca colostrum could be replaced by a bovine [30] or goat one [16]. Walker [19] even suggests that if the premature cria is too weak and does not have a suckle reflex, the colostrum and milk should be given by orogastric intubation. In the present study, the need for bottle feeding was associated with occurrence of more frequent cria losses. It was shown that the percentage of falls within 12 months after birth was higher in the groups of bottle-fed crias. In some authors’ reports [30,31], crias with different health problems were fed by bottle. Some of them did not survive. 

An important finding in the present study was the positive correlation between the birth weight of full-term, preterm, and post-term crias in the surveyed alpaca herds. It was found that post-term crias (born after 370 days of gestation) had higher weight than those born at term. Similar results were reported by Davis et al. [1], where the birth weight increased by 0.05 kg on each additional day of pregnancy. In contrast, Pearson et al. [4] observed an inverse relationship, where post-term crias had lower birth weight than those born at term. Therefore, the rearing conditions and the level of care of dams on the farm are important in this aspect. Probably, better rearing conditions and a higher level of care contribute to the higher birth weight in full-term, preterm, and post-term crias. This is important, as a small cria usually has a lower chance of survival and proper growth [5], and the costs of treatment or care are higher [26]. 

In the present study, only 21 out of the 109 owners reported a twin pregnancy in their herd. As reported by Bravo et al. [32] and Campbell et al. [25], double ovulation in alpacas occurs in 6–10% of cases. However, twin pregnancies are undesirable due to the high risk of miscarriage, which was also reported in the present survey (81% of twin pregnancies ended in miscarriage).

As demonstrated by Pearson et al. [4], 7% to 12% of pregnancies in alpacas end in miscarriage, and the birth rate in South America is estimated at merely 45%. The cria survival rate up to 6 months of age is estimated at 78–91% and is associated with, e.g., the age of the dam and birth weight [5]. Tibary et al. [15] estimated the survival rate at only 67–83%. In the present study, the survival rate of preterm crias before 6 months of age was estimated at 78%. It can be assumed that the survival rate in full-term crias is higher, as only 50% of the surveyed farms recorded animal falls within 12 months after birth in the last 3 years. However, the increased risk of miscarriages and stillbirths in herds with scabies infestations or infectious diseases seems disturbing. It is known that infections by certain bacteria, viruses, fungi, and parasites cause fetal death [28]. Some of such diseases are bovine viral diarrhea (caused by BVDV) [33,34,35], campylobacteriosis (caused by *Campylobacter fetus*) [36], anaplasmosis (caused by *Anaplasma phagocytophilum*) [29], coccidiomycosis (caused by *Coccidioides* spp.) [37], toxoplasmosis (caused by Toxoplasma gondii) [38,39], and neosporosis (caused by *Neospora* spp.) [38,40]. Mixed infections (usually bacterial) have been indicated as the diagnosed causes of miscarriages by Rüfli et al. [41]. It was difficult to identify the main cause in autopsy (most likely the miscarriages were a result of severe stress or a disease being an indirect cause). The present study has shown a higher frequency of miscarriages, stillbirths, and cria deaths within 12 months after birth in herds affected by infectious diseases. As reported by Tibary et al. [15,28], infections, congenital defects, disorders in urine and meconium excretion, and abnormalities in the umbilical area are common causes of cria falls shortly after birth. Moreover, their study demonstrated a relationship between the occurrence of infectious diseases and miscarriages and stillbirths of unknown etiology. It can be presumed that the disease may have caused fetal death, which was difficult to prove unambiguously since no post-mortem examination was performed. This finding was also confirmed by Rüfli et al. [41]. This phenomenon can be considered dangerous, as the alpaca owner may not be aware of the infectious disease, which may be harmless to adult animals but may induce miscarriage and stillbirth, thereby significantly reducing the reproductive success in the herd. In herds with diagnosed scabies infestation, miscarriages and preterm deliveries are reported to occur more frequently. This parasite has no direct effect on the fetus but deteriorates the health of the dam, leading to weight loss [42] and impairment of reproduction performance. The present study revealed a close correlation between miscarriages of unknown etiology and scabies infestations. This is a very disturbing phenomenon, as every third survey respondent indicated the problem of scabies infestation on the farm, and every fifth owner reported cases of infectious diseases in the herd, which can be considered a fairly high percentage.

A positive finding of the survey is the relationship between the supplementation provided to female alpacas and the positive effect on reproductive results. Adequate nutrition is one of the most important factors in animal reproductive success [3]. As reported by the author of [22], the requirement for calcium (Ca) or phosphorus (P) and vitamin A or E increases almost 2–3 times and 1.5–2 times, respectively, during pregnancy. It was found in the present study that the cria birth weight was significantly higher in herds where the dams received mineral supplementation; hence, it can be concluded that the supplementation helps to meet the needs of pregnant females. An adequate supply of trace elements (copper, selenium, zinc) and fat-soluble vitamins (A and E) has a positive effect on the functions of the immune system in both the dam and the cria, which is another important factor related to the survival of young alpacas. It is assumed that the deficiency of these components may lead to a higher frequency of miscarriages and perinatal falls [3].

## 5. Conclusions

It is evident that reproduction performance in alpaca herds is influenced by many factors related to animal rearing conditions, health, and supplementation. The present study has shown factors that may have a negative or positive impact on cria survival and on the occurrence or limitation of reproduction-related problems. An important element increasing the cria survival rate is the birth weight, as its higher value contributes to a lower risk of perinatal complications, eliminates the necessity of bottle feeding, and ensures a greater likelihood of proper growth of these animals. Equally important are the determinants of the occurrence of miscarriages and stillbirths. It is disturbing that a large number of alpaca owners do not identify the cause of fetal death, e.g., through an autopsy, while this study has indicated a relationship between such cases and the occurrence of infectious diseases and scabies infestation in the herd. It can therefore be presumed that they may be potential causes of reproduction-related problems that are not discerned in time. A positive aspect is the confirmation of the relationship between supplementation (mainly mineral) in pregnant females and cria birth weight, which increases the chances of cria survival in the first months after birth.

## Figures and Tables

**Table 1:** Summary of the topics covered by the questionnaire.

Section	Topics
Farm characteristics	The location of the farm (Continental Europe, the British Isles, North America); the time of running the farm (up to 10 years, 11 years, and more); the number of females in the herd (up to 20, 21, and more); the number of stud males (up to 5, 6, and more); the number of crias on the farm (up to 10, 11, and more)
Animal health status	Mineral supplementation in pregnancy (Yes/No); vitamin supplementation in pregnancy (Yes/No); necessity of bottle feeding (Yes/No); the presence of scabies infection in the herd (Yes/No); cases of any infectious (bacterial, viral) diseases in the herd (Yes/No)
Reproductive indicators	Pregnancy diagnosis (Yes/No); birth weight (kg) of crias born at term on the farm; questions about preterm cria (number of cases, days before due date parturition, birth weight (kg) of preterm crias, how many crias lived up to 6 months); questions about post-term crias (number of cases, days after due date parturition, birth weight (kg) of post-term crias, how many crias live up to 6 months); questions about twin pregnancies (number of cases, survivability); number of all miscarriages in the herd in the last 3 years and the causes of miscarriage; number of miscarriages associated with: diseases, high levels of maternal stress, weather-related factors (very low/high temperature, smoke produced by forest fires), and accidents or not known causes; number of all stillbirths in the herd in the last 3 years; number of stillbirths associated with: perinatal problems, genetic defects, dam diseases, or not known causes; number of cria falls within 12 months after birth in the last 3 years; number of cria falls associated with: perinatal problems, genetic defects, dam diseases, accidents or not known causes

**Table 2:** Mean values ($\bar{x}$ ± SD) of cria birth weight taking into account the birth term and reproductive loss rates in the analyzed regions. The * symbol indicates a significant difference at *p* ≤ 0.05, and the *** symbol indicates a significant difference at *p* ≤ 0.001.

	British Isles	Continental Europe	North America	Chi2	*p*-Value
Cria birth weight (kg)	7.86 ± 0.88 N = 22	7.42 ± 0.84 N = 31	7.34 ± 0.87 N = 56	8.541	0.014 *
Birth weight of premature cria (kg)	6.64 ± 1.45 N = 14	5.94 ± 1.45 N = 18	4.53 ± 1.24 N = 20	15.869	0.0004 ***
Birth weight of postmature cria (kg)	8.47 ± 1.37 N = 17	8.09 ± 1.16 N = 19	8.04 ± 1.24 N = 28	1.339	0.512
Cases of miscarriage (n)	1.05 ± 1.46 N = 22	1.06 ± 1.5 N = 31	1.02 ± 1.74 N = 56	0.218	0.897
Cases of stillbirth (n)	0.45 ± 0.67 N = 22	0.48 ± 0.85 N = 31	0.45 ± 0.63 N = 56	0.102	0.95
Deaths up to 12 months (n)	1.32 ± 2.38 N = 22	1.29 ± 1.62 N = 31	1.5 ± 3.88 N = 56	0.624	0.732

**Table 3:** Effect of supplementation of pregnant females on cria birth weight taking into account the birth term ($\bar{x}$ ± SD) and reproductive loss rates ($\bar{x}$ ± SD). The * symbol indicates a significant difference at *p* ≤ 0.05.

	Mineral Supplementation	No Mineral Supplementation	*p*-Value	Vitamin Supplementation	No Vitamin Supplementation	*p*-Value
Cria birth weight (kg)	7.58 ± 0.9 N = 81	7.16 ± 0.75 N = 28	0.0198 *	7.52 ± 0.8 N = 65	7.4 ± 0.99 N = 44	0.175
Birth weight of premature cria (kg)	5.61 ± 1.74 N = 34	5.53 ± 1.29 N = 15	0.9	5.82 ± 1.62 N = 37	5.0 ± 1.49 N = 15	0.107
Birth weight of postmature cria (kg)	8.33 ± 1.23 N = 44	7.8 ± 1.22 N = 20	0.057	8.24 ± 1.2 N = 39	8.04 ± 1.34 N = 25	0.286
Cases of miscarriage (n)	0.95 ± 1.42 N = 81	1.29 ± 2.07 N = 28	0.839	1.0 ± 1.48 N = 65	1.09 ± 1.8 N = 44	0.814
Cases of stillbirth (n)	0.47 ± 0.71 N = 81	0.43 ± 0.69 N = 28	0.743	0.52 ± 0.77 N = 65	0.36 ± 0.57 N = 44	0.378
Deaths up to 12 months (n)	1.44 ± 3.28 N = 81	1.29 ± 2.45 N = 28	0.419	1.23 ± 1.82 N = 65	1.66 ± 4.33 N = 44	0.638

**Table 4:** Impact of infectious diseases on cria birth weight taking into account the birth term ($\bar{x}$ ± SD) and reproductive loss rates ($\bar{x}$ ± SD). The * symbol indicates a significant difference at *p* ≤ 0.05, and the *** symbol indicates a significant difference at *p* ≤ 0.001.

	No Infectious Diseases in Farm in the Last 3 Years	Infectious Diseases in Farms Occurred in the Last 3 Years	*p*-Value
Cria birth weight (kg)	7.35 ± 0.8 N = 85	7.92 ± 1.0 N = 24	0.017 *
Birth weight of premature cria (kg)	5.54 ± 1.64 N = 37	5.7 ± 1.59 N = 15	0.617
Birth weight of postmature cria (kg)	8.06 ± 1.21 N = 47	8.44 ± 1.33 N = 17	0.557
Cases of miscarriage (n)	0.85 ± 1.61 N = 85	1.71 ± 1.45 N = 24	0.0006 ***
Cases of stillbirth (n)	0.33 ± 0.56 N = 85	0.92 ± 0.93 N = 24	0.0009 ***
Deaths up to 12 months (n)	0.99 ± 1.61 N = 85	2.88 ± 5.67 N = 24	0.019 *

## Data Availability

The data presented in this study are available on reasonable request from the corresponding author.

## Supplementary Materials

The following supporting information can be downloaded at: https://www.mdpi.com/article/10.3390/ani12213030/s1, File S1: Questionnaire Form.
