# Supplementary material for: Reproductive Losses and Their Causes in Alpacas—A Survey-Based Study
Source: Animals (Basel). 2022 Nov 3;12(21):3030. doi: 10.3390/ani12213030 (PMC9657741; doi:10.3390/ani12213030)
Supplement: Supplementary file 1 [file animals-12-03030-s001.zip › animals-1983874-supplementary.pdf]

Supplementary material S1 – Questionnaire Form

The questionnaire is dedicated to alpacas' breeders. It concerns the issue of parturition, the frequency of stillbirth and twin pregnancies. We will be very grateful for your responses. Collected data will be used only for scientific purposes. The questionnaire was available in Google Forms and is completely anonymous.

Part 1 – Farm characteristic

In this part of the questionnaire please give the information about your farm.

1. In which country is your farm located?*

………………………………………………….

2. How long do you breed alpacas?*

- ☐ < 10 years
- ☐ >10 years

3. How many dams (females) do you have on the farm?*

- ☐ < 20
- ☐ >21

4. How many stud males do you have on the farm?*

- ☐ < 5
- ☐ > 6

5. How many crias (youngs to 6 month of life) do you have on the farm?*

- ☐ <10
- ☐ >11

6. What other animals live on the farm?*

- ☐ None
- ☐ Horses
- ☐ Cattle
- ☐ Sheep
- ☐ Goats
- ☐ Dogs
- ☐ Cats
- ☐ Rabbits
- ☐ Other:………………………….

Part 2 – Animal health status

In this section, please give the information about your animals' health status.

7. Do pregnant females receive mineral supplementation?*

- ☐ Yes
- ☐ No

8. Do pregnant females receive vitamin supplementation?*

- ☐ Yes
- ☐ No

9. Have you ever had to feed cria from the bottle?*

- ☐ Yes
- ☐ No

10. Have ever been any cases of sarcoptic mange on your farm in the last 3 years?*

- ☐ Yes
- ☐ No

11. Have ever been any cases of infectious (viral, bacterial) or parasitic diseases on your farm in the last 3 years? You can select more than one answer.*

- ☐ Bovine Viral Diarrhea Virus (BVDV)
- ☐ Brucella spp (Brucelosis)
- ☐ Camplyobacter fetus (or spp)
- ☐ Chlamydophila abortus (or spp)
- ☐ Leptospira spp (leptospirosis)
- ☐ Listeria monocytogenes (listeriosis)
- ☐ Salmonella spp (salmonellosis)
- ☐ Trueperella pyogenes
- ☐ Neospora caninum
- ☐ Toxoplasma gondii (toxoplasmosis)
- ☐ Equine viral arteritis (EAV)
- ☐ Mycoplasma
- ☐ Escherichia coli
- ☐ Anaplasma phagocytophilum (anaplasmosis)
- ☐ Not occured/ Not diagnosed
- ☐ Other:……………………………………

Part 3 – Reproductive indicators

In this section, please give the information about parturition in your farm (single/twin), if stillbirths or miscarriages occurred.

12. Do you diagnose pregnancy in your farm?*

- ☐ Yes
- ☐ No

13. What is the average birth weight of cria in your farm?*

………………………………………

14. Have ever been any premature births in the farm within the last 3 years?*

- ☐ Yes
- ☐ No

15. If yes, how many such cases have there been?

……………………………………..

16. If yes, how long before due date the cria was born (in days)?

……………………………………..

17. What was birth weight of premature newborn?

………………………………………

18. How many premature newborn live up to 6 month of life?

………………………………………

19. Has ever been any postmature newborn in the farm within the last 3 years (parturition after due date)? *

- ☐ Yes
- ☐ No

20. If yes, how many such cases have there been?

……………………………………..

21. If yes, how long after due date was the cria born (in days)?

………………………………………

22. What was birth weight of postmature newborn?

………………………………………

23. How many postmature newborn lived to 6 month of life?

………………………………………

24. Has there ever been a twin pregnancy in your farm?*

- Yes
- No

25. If yes, how many such cases have there been?

…………………………………….

26. Were the twins born alive?

- Yes
- No, born on due date, but dead (stillbirth)
- No, born dead before due date (miscarriage)
- Other:………………………………..

27. If twins were born alive, did both lived to 6 month of life?

- Yes
- No, only one lived
- No, none lived

28. In the last 3 years, have any miscarriages (only in single pregnancies) occurred?

- Yes
- No

29. If yes, how many such cases have there been?

…………………………………….

30. What was the cause of miscarriage (e.g. mothers' strong stress, parasitic / viral/ bacterial disease, unknown)?

……………………………………..

31. In the last 3 years, have any stillbirths occurred in your farm (parturition on due date, but cria was dead)?

- Yes
- No

32. If yes, how many such cases have there been?

……………………………………

33. What was the cause of stillbirth (e.g. parasitic / viral / bacterial disease – what kind, parturition problems - wrong fetus position, cesarean section, unknown)?

…………………………………

34. In the last 3 years, have ever any cria falls occurred in your farm to 12 months after giving birth?

- Yes
- No

35. If yes, how many such cases have there been?

……………………………………

36. What was the cause of cria falls (e.g. parasitic / viral / bacterial disease – what kind, congenital defects, mother' lactation problems, accident, unknown)?

…………………………………
